# MEG3 promotes proliferation and inhibits apoptosis in osteoarthritis chondrocytes by miR-361-5p/FOXO1 axis

**DOI:** 10.1186/s12920-019-0649-6

**Published:** 2019-12-30

**Authors:** Anying Wang, Naixia Hu, Yefeng Zhang, Yuanzhen Chen, Changhui Su, Yao Lv, Yong Shen

**Affiliations:** 1 Doctor Student, Hebei Medical University, No. 361, Zhongshan East Road, Hebei Province, Shijiazhuang, 050017 China; 2 Department of Orthopedic, The Second Affiliated Hospital of Shandong First Medical University, No. 366, Taishan Street, Shandong Province, Taishan, 271000 China; 3 ICU, The Second Affiliated Hospital of Shandong First Medical University, No. 366, Taishan Street, Shandong Province, Taishan, 271000 China; 4 Department of Orthopedic, The Central Hospital of Taian City, No. 29, Longtan Road, Shandong Province, Taian, 271000 China; 5 Department of Orthopedic, The Third Hospital of Hebei Medical University, No. 139, Ziqiang Road, Hebei Province, Shijiazhuang, 050051 China

**Keywords:** Osteoarthritis, Maternally expressed 3, miR-361-59, FOXO1, ECM degradation

## Abstract

**Background:**

This study aimed to investigate the role of long non-coding RNA (lncRNA) maternally expressed 3 (MEG3) and related molecular mechanisms, in osteoarthritis (OA).

**Methods:**

Cartilage tissues of OA patients and healthy volunteers were isolated and cultured. After transfection with the appropriate constructs, chondrocytes were classified into Blank, pcDNA3.1-NC, pcDNA3.1-MEG3, si-NC, si-MEG3, pcDNA3.1-NC + mimics NC, pcDNA3.1-MEG3 + mimics NC, pcDNA3.1-NC + miR-361-5p mimics and pcDNA3.1-MEG3 + miR-361-5p mimics groups. qRT-PCR was used to detect the expression of MEG3, miR-361-5p and FOXO1*.* Western blot, luciferase reporter assay, RIP, CCK-8, and flow cytometry analysis were performed to reveal the morphology, proliferation, and apoptotic status of cartilage cells. Histological analysis and immunostaining were conducted in the OA rat model.

**Results:**

Expression of MEG3 and FOXO1 was significantly decreased in OA compared with the normal group, while the expression of miR-361-5p was increased. MEG3 might serve as a ceRNA of miR-361-5p in OA chondrocytes. Moreover, using western blot analyses and the CCK-8 assay, MEG3 was shown to target miR-361-5p/FOXO1, elevate cell proliferation, and impair cell apoptosis. Functional analysis in vivo showed that MEG3 suppressed degradation of the cartilage matrix.

**Conclusion:**

MEG3 can contribute to cell proliferation and inhibit cell apoptosis and degradation of extracellular matrix (ECM) via the miR-361-5p/FOXO1 axis in OA chondrocytes.

## Background

Osteoarthritis (OA) is a common condition whose prevalence and severity increase with age and which can induce serious pain and disability [1]. OA is characterized by various pathological changes, such as articular cartilage degradation, synovial inflammation and subchondral osteoblast activation [2]. Although various genetic, biological, and biomechanical components have been proven to be associated with OA [3], the underlying molecular mechanisms of OA progression remain unclear and an efficacious cure is still not available.

Several recent reports have implicated lncRNAs in various cancerogenesis-related signalling pathways [4–8]. LncRNAs have also been reported to be associated with OA progression in the knee [9]. The lncRNA maternally expressed 3 (MEG3) has been shown to be an important factor in tumour development [10]. However, in addition to its role in the development of multiple cancers including lung cancer [11], breast cancer [12] and esophageal cancer [12], MEG3 has been found to be a potential therapeutic target for OA. Su et al. demonstrated that levels of MEG3 were dramatically decreased in OA and inversely related to levels of vascular endothelial growth factor A [13]. You et al. provided evidence that MEG3 suppressed chondrogenic differentiation of synovium-derived mesenchymal stem cells by epigenetically hindering TRIB2. Methylene blue has been shown to slow the progression of OA by regulating the expression level of MEG3 [14]. Down-regulation of MEG3 facilitated the development of OA via the miR-16/SMAD7 axis [15]. Jin et al. demonstrated that down-regulation of MEG3 was associated with OA progression through a miRNA-target gene axis [15]. On the basis of the research listed above, we concluded that MEG3 might be involved in OA development via multiple signal axes.

This study was devised in order to identify novel molecular mechanisms of MEG3 action and to complement present understanding of the MEG3-centric regulatory network in OA. In this study, the role of MEG3 and related molecules in OA were explored using cartilage cells obtained from patients with OA (OA group) or from patients undergoing artificial joint replacement (normal group). Through our in vitro and in vivo experiments, we found that MEG3 could promote chondrocyte proliferation, impair apoptosis, and eliminate ECM degradation in OA via miR-361-5p/FOXO1. These results provide new insights into the molecular mechanisms of OA progression and identify MEG3/miR-361-5p/FOXO1 as a novel therapeutic candidate for the treatment of OA.

## Methods

### Patients and grouping

The cartilage tissues were obtained from the knee joints of 30 patients who underwent total knee arthroplasty. Meanwhile, the healthy cartilage tissues were collected from 20 patients without OA or RA (rheumatoid arthritis). There was no statistical difference in age and gender between OA patients and healthy volunteers. The age and sex of all the samples were matched. All patients voluntarily signed a notice of informed consent. The current study was approved by the Ethics Committee of the hospital (ethic vote 198/203).

### Cell culture

The femoral articular cartilage of the knee joint was harvested and the tissue was digested with 0.2% type II collagenase for 40 min at 37 °C. The isolated cells were washed with D-Hanks solution and suspended in DMEM/F12 (Gibco, USA) containing 10% FBS, 100 U/mL penicillin (Gibco, USA) and 100 mg/mL streptomycin (Gibco, USA). The chondrocytes were then cultured in a high humidity incubator (37 °C; 5% CO_2_). The media were refreshed every two days until the chondrocytes had grown into sheets and were over 85% confluent [16]. After 2 or 3 passages, chondrocytes were used for furthering our investigations.

### Cell transfection

The MEG3 overexpression vector pcDNA3.1-MEG3, si-MEG3 for MEG3 knockdown, si-FOXO1 used to decrease the expression of FOXO1 (Sangon Biotech, Shanghai, China), miR-361-5p mimics, and miR-361-5p mimics NC (Guangzhou Reeber) were used to transfect chondrocytes using the Lipfectamine 2000 transfection kit according to the manufacturer’s instructions (Invitrogen, USA). After 48 h, the samples were treated with IL-1β (10 ng/mL) for 24 h. Cells of each group were then collected for experimentation.

### Real-time fluorescent quantitative PCR

Total RNA from each sample was isolated using TRIzol reagent. Using 500 ng of total RNA, cDNA template was synthesized by PrimeScript RT kit (Takara biomedical Technology Co., Ltd., Beijing, China). GAPDH was used as the reference gene (Table 1). The qRT-PCR conditions were as follows: initial denaturation at 95 °C for 3 min, then 39 cycles of denaturation at 95 °C for 10 s and annealing at 55 °C for 45 s, and 72 °C extension for 10 s. qRT-PCR was performed on the ABI7500 platform Relative expression of the candidate genes were calculated using the 2^-∆∆CT^ method [17].

**Table 1:** The amplification primer used for current RT-qPCR analysis

Name of primer	Sequences
LncRNA MEG3	5′-CTGCCCATCTACACCTCACG-3′
	5′-CTCTCCGCCGTCTGCGCTAGGGGCT-3′
GAPDH	5′-TGCACCACCAACTGCTTAGC-3′
	5′-GGCATGCACTGTGGTCATGAG-3′
miR-361-5p	5′-ATAAAGTGCTGACAGTGCAGATAGTG-3′
	5′-TCAAGTACCCACAGTGCGGT-3′
U6	5′-CTCGCTTCGGCAGCACA-3′
	5′-AACGCTTCACGAATTTGCGT-3′
FOXO1	5′-GAGGAGCCTCGATGTGGATG-3
	5′-CCGAGATTTGGGGGAACGAA-3

Notes: *MEG3* maternally expressed 3, *GAPDH* glyceraldehyde-3-phosphate dehydrogenase, *RT-qPCR* real-time fluorescence quantitative polymerase chain reaction

### Luciferase reporter assay

A regulatory relationship between MEG3 and miR-361-5p was predicted using StarBase; likewise, a relationship between miR-361-5p and FOXO1 was predicted. A fragment comprising the putative binding site between MEG3 3’UTR and miR-361-5p (MEG3-WT) was cloned into the pmirGLO reporter vector (Promega, USA).A second construct without the above fragment (MEG3-MUT) was also created. Human chondrocytes were seeded in 24-well plates (5 × 10^5^/well) and incubated for 24 h., miR-361-5p mimic or mimics NC, and MEG3-WT and MEG3-MUT were co-transfected into human chondrocytes by Lipofectamine 3000 (Thermo Fisher Scientific), and a dual-luciferase reporter gene assay was performed after 48 h transfection. Likewise, FOXO1-WT and FOXO1-MUT were synthesized, and then, the correlation of miR-361-5p and FOXO1 expression was determined using the dual-luciferase reporter gene assay.

### RNA immunoprecipitation (RIP) assay

RIP was performed using a Magna RIPTM RNA kit (Millipore, USA). Briefly, cultured chondrocytes were suspended in RIP lysis buffer (Solarbio) and incubated in RIP buffer containing human anti-Ago2 antibody beads (Millipore) overnight (Input and normal IgG served as controls). Next, RNAs were extracted using TRIzol reagent to follow the relative enrichment of MEG3/FOXO1 and miR-361-5p.

### Western blotting

Chondrocytes were lysed with RIPA buffer to extract the whole proteins. The total protein content of the extracted samples was quantified with a bicinchoninic acid (BCA) protein assay. Denatured samples containing equal amounts of protein (50 μg) were separated on 10% polyacrylamide gels and transferred to polyvinylidenefluoride membranes. The membranes were blocked (5% Skim milk/BSA) and incubated with primary antibodies including: β-Catenin (#19807), MMP-13 (#94808), Collagen II (#34712), PCNA (#13110), Bax (#5023), Bcl-2 (#4223) antibody (1:1000, Cell Signaling, Boston, USA), as well as Ki67 (ab92742), ADAMTS-5 (ab41037) and Aggrecan (ab36861) (1: 1000, Abcam, Cambridge, MA, USA) [15]. After washing, the membranes were incubated with HRP-conjugated secondary antibody (1:5000, #7074; Cell Signaling Technology, Danvers, MA, USA). Finally, the protein was developed with diaminobenzidine (DAB). All experiments were repeated 3 times.

### CCK-8 assay

Cells were transferred to 96-well plates (2 × 10^3^ cells/well) and cultured for 0, 24, 48, and 72 h (at 37 °C and 5% CO_2_). 10 μL of CCK-8 (Sigma-Aldrich) reagent was added into each well and the plates were incubated for an additional 2 h. Finally, the OD_490_ value was measured by enzyme-linked immunosorbent assay [18].

### Flow cytometry assay

Cells were treated with trypsin supplemented with 200 μL Annexin V-FITC, then incubated for 10 min in the dark. Cells were then washed with 200 μL PBS and 10 μL PI was added. Cell apoptosis was detected by flow cytometry (Beckman Coulter.

### OA rat model construction

A total of 20 male SD rats (200–250 g; five rats in each group) were obtained from the Experimental Animal Center of Taishan Medical College. Before starting any intervention, all rats were allowed to acclimate in the SPF animal facility with a 12 h light-dark cycle for one week (24–26 °C; 50–60% humidity). The rats were fed a commercial pellet diet (Niroo Sahand, Tabriz, Iran) and sterile drinking water At the beginning of the procedure, all rats were anesthetized with an intramuscular injection of sodium pentobarbital (0.05 mg/g, Chuangdong Co., Chongqing, China). SD rats were traversed by the medial collateral ligament and destabilized by the medial meniscus (DMM). One week after the operation, si-NC and si-MEG3 (1 × 10^9^ PFU, 20 μL) were injected into the knee joint of the recipient rat (20 μL per joint) twice a week for 4 weeks (*n* = 5 for each group). Eight weeks after the operation, the rats were sacrificed by the cervical dislocation method, and the knee joints were harvested. All experiment was performed in the Experimental Center of Taishan Medical College. This study was approved by the Laboratory Animal Ethics Committee of Taishan Medical College (No. 2019146), and all experiments abided by the Guide for the care and use of laboratory animals.

### Histological and immunostaining analyses

Cartilage samples were fixed in paraformaldehyde (4%), embedded in paraffin, and cut into sections (5 μm/slice). The cartilage destruction was evaluated using the Safranin ‘O’ staining protocol. Histological scores were assessed according to the International Osteoarthritis Research Association (OARSI) grading system, which ranges from 0 (normal) to 6 (> 80% cartilage loss). Scores were determined from multiple serial sections of the knee joint of each rat.

### Statistical analysis

SPSS 18.0 (Chicago, IL, USA) software was used for data analysis. All the data in current investigation are presented as the mean ± standard deviation (SD). Significant differences between two groups were assessed using a Student’s t-test. A one-way ANOVA followed by the least significant difference between means (LSD)-t multiple comparison test was used for comparing more than two groups. Spearman analyses were performed to identify the correlations of miR-361-5p and MEG3 or FOXO1. *P* < 0.05 was considered as statistically significant.

## Results

### MEG3 regulated OA cell proliferation, apoptosis, and cartilage matrix degradation

**Fig. 1:**
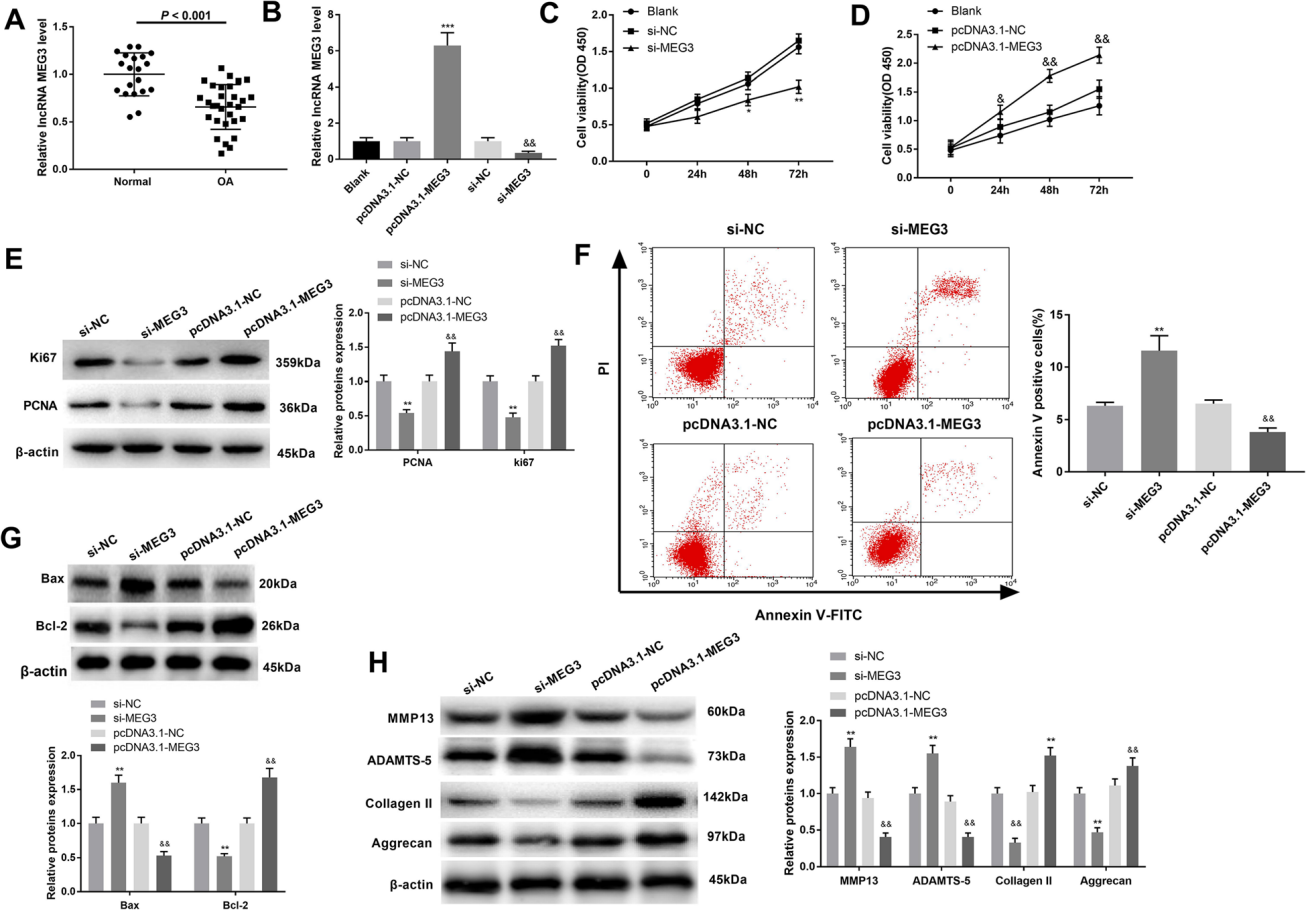
The effect of MEG3 in OA chondrocyte proliferation, apoptosis and cartilage matrix degradation. **a**, the expression of MEG3 in normal group and OA group detected by qRT-PCR; data expressed as mean ± standard deviation (SD). **b**, expression of MEG3 by qRT-PCR after transfection of OA chondrocytes with pcDNA3.1-MEG3 or si-MEG3. **c**, CCK-8 showed the proliferation of cells transfected with si-MEG3; MEG3 knockdown inhibited the proliferation of OA chondrocytes. **d**, the proliferation of cells after pcDNA3.1-MEG3 transfection detected by CCK-8 assay. **e**, the expression of PCNA and marker Ki67 was detected by western blot after transfection with pcDNA3.1-MEG3 or si-MEG3 in OA chondrocytes. **f**, cell apoptosis of OA chondrocytes was detected by flow cytometry. **g**, the expression of apoptosis-related proteins Bcl-2 and Bax in OA chondrocytes was measured by western blot; **h**, Western blot was performed to assess the expression of cartilage matrix proteins (MMP13, ADAMTS-5, Collagen II, Aggrecan) in chondrocytes of each group. *, *P* < 0.05 compared with Blank or pcDNA3.1-NC group. & *P* < 0.05, compared with the Blank or si-NC group

Within comparison to the normal group, MEG3 in chondrocytes of the OA group was significantly decreased (*P* < 0.001) (Fig. 1a). Expression of MEG3 in the pcDNA3.1-MEG3 group was dramatically increased compared with the pcDNA3.1-NC group (Fig. 1b) (*P* < 0.05). Similarly, MEG3 expression in the si-MEG3 group was significantly suppressed compared to the si-NC group (Fig. 1b) (*P* < 0.05). MEG3 silencing significantly inhibited cell proliferation compared with si-NC (Fig. 1c) (*P* < 0.05). In contrast, the cell proliferation ability of the pcDNA3.1-MEG3 group was greatly elevated compared to the pcDNA3.1-NC group (Fig. 1d) (*P* < 0.05). Western blot analyses with the proliferative markers Ki67 and PCNA showed results consistent with the CCK-8 analysis (Fig. 1e) (*P* < 0.01). In chondrocytes transfected with si-MEG3, apoptosis was dramatically elevated compared with the si-NC group, whereas overexpression of MEG3 inhibited apoptosis of chondrocytes (Fig. 1f) (*P* < 0.01). These results were confirmed by western blot analysis. Thus, the apoptotic protein Bax was significantly increased after MEG3 knockdown, and attenuated by elevated MEG3 expression. Conversely, the anti-apoptosis protein Bcl-2 revealed the opposite tendency (Fig. 1g) (*P* < 0.01).

Analysis of the rat OA model showed that MMP13 and ADAMTS-5 were up-regulated in the si-MEG3 group (compared with si-NC group), while the expression of Collagen II and Aggrecan were down-regulated (Fig. 1h) (*P* < 0.01). Conversely, MEG3 overexpression drastically reduced the expression levels of MMP13 and ADAMTS-5, while expression of Collagen II and Aggrecan were enhanced (Fig. 1h) (*P* < 0.01). Together, these results demonstrate that MEG3 can promote proliferation, inhibit apoptosis, and reduce ECM degradation in OA.

### MEG3 acts as a ceRNA of miR-361-5p in OA chondrocytes

**Fig. 2:**
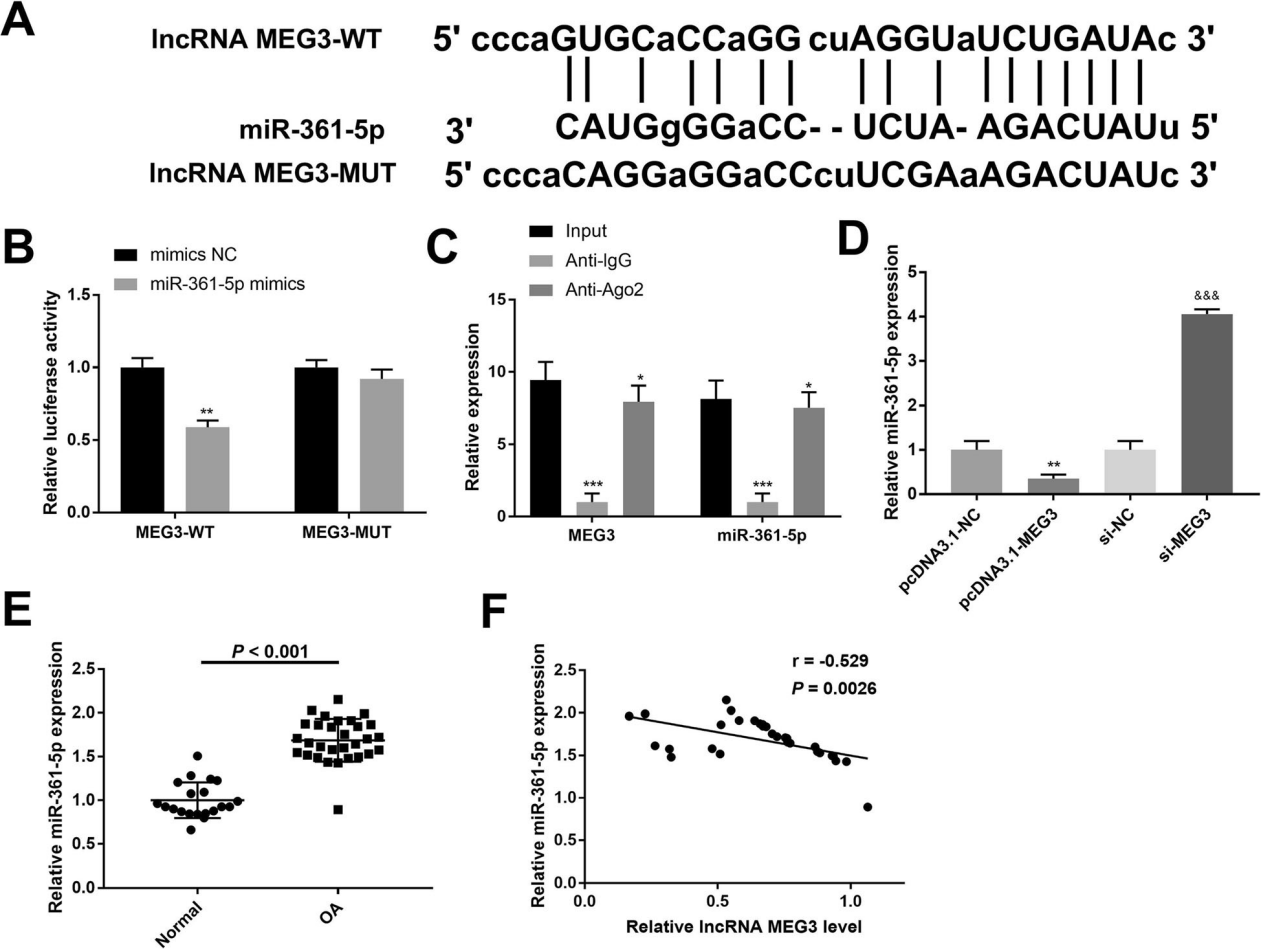
MEG3 is a ceRNA of miR-361-5p in OA chondrocytes **a**, the binding sites of MEG3 and miR-361-5p predicted by bioinformatics analysis. **b**, luciferase reporter gene assay showed that the microRNA-361-5p mimic reduced the luciferase activity of MEG3-Wt in chondrocytes. **c**, the concentration of MEG3 and miR-361-5p in IgG or Ago2 immunoprecipitates detected by RIP and qRT-PCR. **d** and **e**, the expression of miR-361-5p in OA tissues or cells was examined by qRT-PCR. **f**, Spearman analysis determined that miR-361-5p was negatively associated with MEG3; *, *P* < 0.05 compared with Blank group or pcDNA3.1-NC group. &, *P* < 0.05 compared with si-NC group. Data expressed as mean ± standard deviation (SD)

The binding site of MEG3 and miR-361-5p was predicted using StarBase (Fig. 2a). The results of the luciferase reporter assay revealed that co-transfection of MEG3-WT and miR-361-5p mimic significantly reduced luciferase activity in chondrocytes while no effect of miR-361-5p mimic was found on the luciferase activity after co-transfection with the MEG3-MUT group (Fig. 2b) (*P* < 0.01). Meanwhile, the RIP assay demonstrated an enrichment of MEG3 and miR-361-5p in the Ago2 pellet (Fig. 2c). qRT-PCR revealed that miR-361-5p was down-regulated in response to up-regulation of MEG3 expression and significantly up-regulated after down-regulation of MEG3 expression (Fig. 2d) (*P* < 0.01). miR-361-5p expression was dramatically increased in chondrocytes of OA compared with the normal group (Fig. 2e) (*P* < 0.001). In addition, Spearman correlation analysis showed a negative correlation between expression of MEG3 and miR-361-5p (*r* = − 0.529, *P* = 0.0026) (Fig. 2f). Thus, we hypothesized that MEG3 acts as a ceRNA of miR-361-5p in OA and negatively regulate the expression of miR-361-5p.

### The effect of MEG3 on proliferation, apoptosis and cartilage matrix degradation of OA chondrocytes was reversed by miR-361-5p

**Fig. 3:**
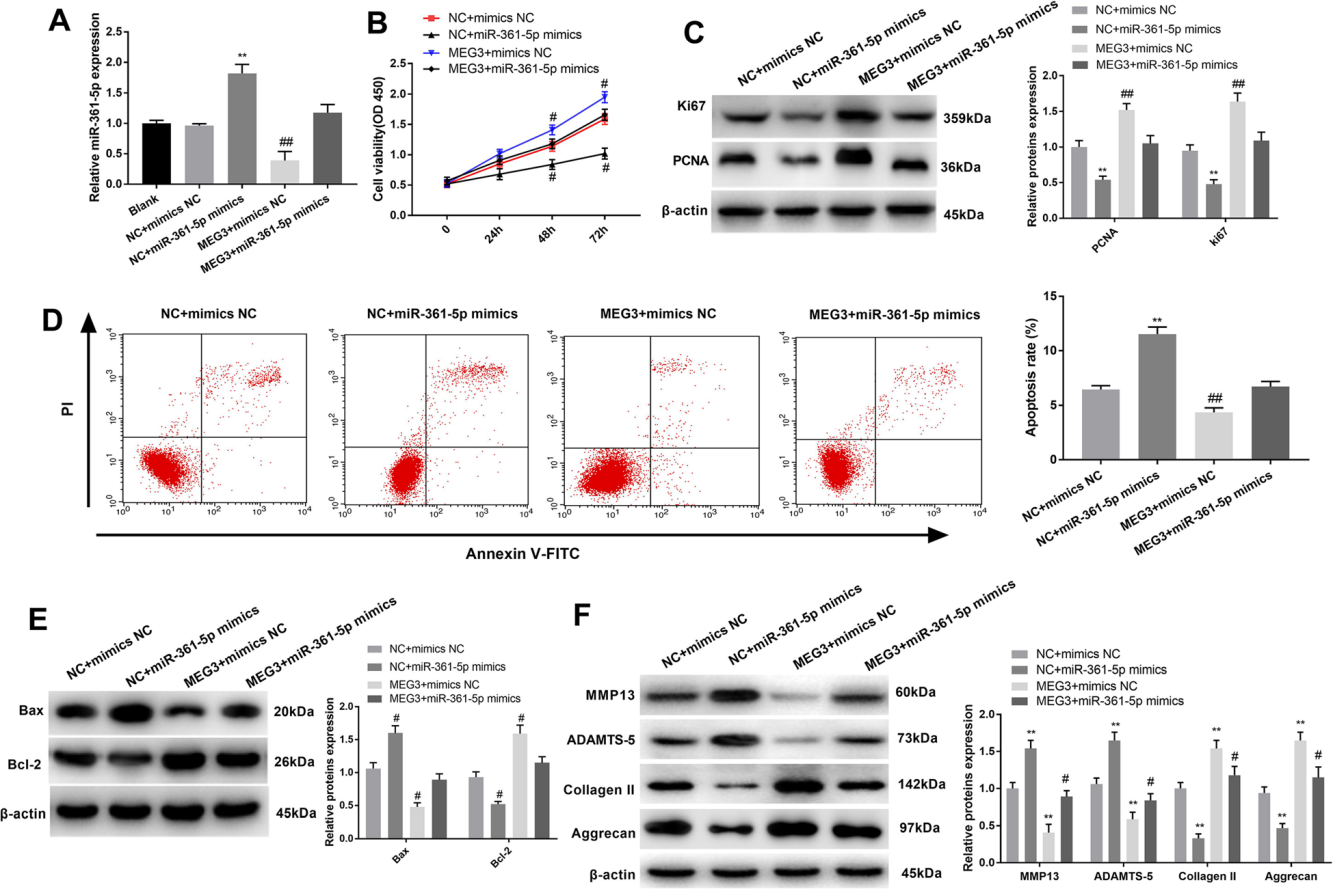
The effect of miR-361-5p on MEG3 in OA chondrocytes. **a**, qRT-PCR detection of miR-361-5p expression. **b**, cell proliferation detected by CCK-8 assay. **c**, detection of PCNA, Ki67 expression by western blot. **d**, flow cytometry was used to detect the apoptosis of OA chondrocytes in each group. **e**, the expression of apoptotic proteins Bcl-2 and Bax detected by Western Blot. **f**, Western blot analysis of cartilage matrix protein expression in chondrocytes of each group. *, *P* < 0.05 compared with Blank group or NC + mimics NC group. ^#^, *P* < 0.05 compared with MEG3 + mimics NC group

To assess the correlation between MEG3 and miR-361-5p in OA, rescue experiments were implemented. qRT-PCR analysis revealed that miR-361-5p was decreased in pcDNA3.1-MEG3 + mimics NC group when compared with pcDNA3.1-NC + mimics NC group, while miR-361-5p was promoted in pcDNA3.1-NC + miR-361-5p mimics group (Fig. 3A) (*P* < 0.01). CCK-8 assay showed that cell viability decreased following overexpression of miR-361-5p. The promoting effect of MEG3 on cell proliferation was reversed by the miR-361-5p mimic (Fig. 3b) (*P* < 0.05). Western blot analyses of PCNA and Ki67 expression were used to verify the results of the CCK-8 assay (Fig. 3c) (*P* < 0.01). Flow cytometry analysis revealed that miR-361-5p inhibited apoptosis and addition of miR-361-5p to the cells transfected with MEG3 (pcDNA3.1-MEG3) promoted cell apoptosis. (Fig. 3d) (*P* < 0.01). As expected, changes in the expression levels of Bax and Bcl-2 were consistent with the flow cytometry data (Fig. 3e) (*P* < 0.05). In addition, the observed decrease in the expression of ECM degradation related proteins, including MMP13, ADAMTS-5, Collagen II, and Aggrecan, indicated that co-transfection of MEG3 and miR-361-5p mimics relieved the ECM degradation that was induced by MEG3 (Fig. 3f) (*P* < 0.05). In summary, these findings suggested that miR-361-5p can reverse the impacts of MEG3 on OA.

### FOXO1 is a direct target of miR-361-5p and positively regulated by MEG3

**Fig. 4:**
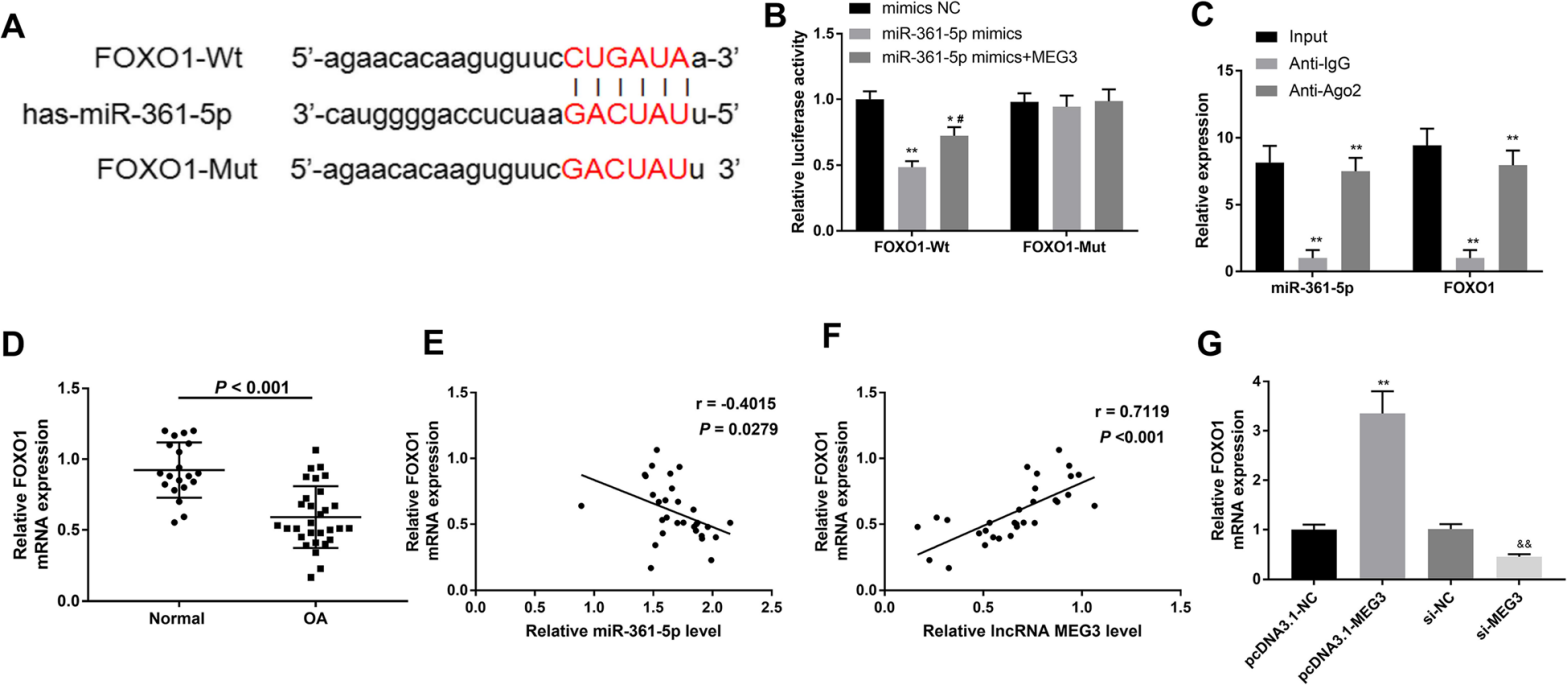
FOXO1 is a directly target of miR-361-5p and positively regulated by MEG3. **a**, the putative binding site between miR-361-5p and FOXO1. **b** and **c**, dual-luciferase reporter gene assay and RIP analysis were performed to verify the correlation of miR-361-5p and FOXO1. **d**, FOXO1 expression was significantly decreased in OA. **e** and **f**, Spearman analyses for the relationships of FOXO1 and miR-361-5p/MEG3. **g**, qRT-PCR revealed that FOXO1 expression was promoted by pcDNA3.1-MEG3 while attenuated by si-MEG3. *, *P* < 0.05 compared with Blank group or pcDNA3.1-NC group. ^#^, *P* < 0.05 compared with miR-361-5p mimics. ^&^, *P* < 0.05 compared with si-NC group

Bioinformatics analysis showed that the putative target of miR-361-5p was FOXO1. A sequence motif at the binding site between miR-361-5p and FOXO1 is exhibited in Fig. 4a. The results of the dual-luciferase reporter assay showed that miR-361-5p significantly decreased the luciferase activity of the FOXO1-WT group, and that up-regulated MEG3 relieved the inhibitory effect induced by miR-361-5p. However, there were no differences in the FOXO1-MUT group (Fig. 4b) (*P* < 0.05). We next verified the associations between miR-361-5p and FOXO1 by conducting a RIP assay. As shown in Fig. 4c, miR-361-5p and FOXO1 could be precipitated by the anti-IgG in contrast to anti-Ago2 (*P* < 0.01). In addition, FOXO1 expression was significantly decreased in OA cartilage tissues compared with normal control (Fig. 4d) (*P* < 0.001), which was similar to the expression pattern of MEG3. Spearman analyses elucidated that FOXO1 was negatively regulated by miR-361-5p (*r* = − 0.4015, *P* = 0.0279) (Fig. 4e), while and positively modulated by MEG3 (*r* = 0.7119, *P* < 0.001) (Fig. 4f). Subsequently, FOXO1 mRNA expression was investigated in OA chondrocytes after MEG3 and si-MEG3 transfections using qRT-PCR. The data showed that overexpression of MEG3 induced FOXO1 expression while interference via si-MEG3 induced the down-regulation of FOXO1 (Fig. 4g) (*P* < 0.01). Collectively, these results revealed that FOXO1 has a close correlation with miR-361-5p and MEG3.

### FOXO1 contributed to the impacts of MEG3 in OA

**Fig. 5:**
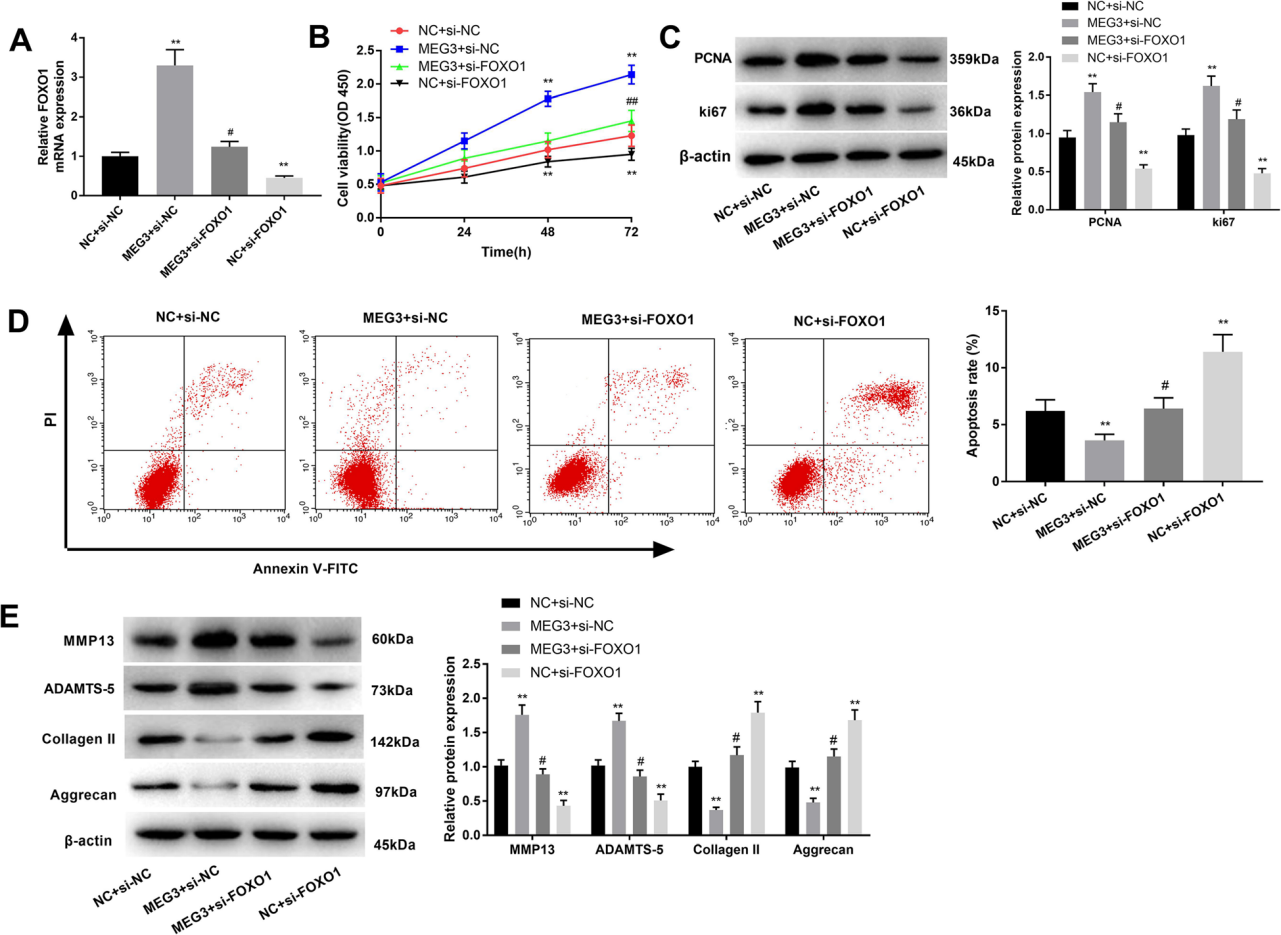
FOXO1 contributed to the impacts of MEG3 in OA. **a**, qRT-PCR showed the FOXO1 expression after diverse treatments. **b** and **c**, CCK-8 and western blot analyses were conducted to explore cell proliferation. **d**, cell apoptosis was determined using flow cytometry. **e**, the effects of FOXO1 on ECM degradation were examined by using western blotting. *, *P* < 0.05 compared with NC + si-NC. ^#^, *P* < 0.05 compared with MEG3 + si-NC

To assess whether FOXO1 affects the function of MEG3 on cell viability, apoptosis and ECM degradation in OA chondrocytes, the latter were transfected with MEG3, MEG3 + si-FOXO1, si-FOXO1, and the corresponding control. qRT-PCR analysis showed that MEG3 could elevate the expression levels of FOXO1 (Fig. 5a) (*P* < 0.05). CCK-8 assay revealed that down-regulation of FOXO1 significantly suppressed cell viability and upon addition of FOXO1in OA chondrocytes with high-regulated MEG3 cell viability was attenuated compared with the MEG3 + si-NC group (Fig. 5b) (*P* < 0.01). PCNA and Ki67 expressions were also inhibited by si-FOXO1, and these decreased the promoting effects of MEG3 (Fig. 5c, *P* < 0.05). Flow cytometry results showed that si-FOXO1 facilitated apoptosis compared with MEG3 + si-NC and MEG3 + si-FOXO1 groups (Fig. 5d) (*P* < 0.05). In the NC + si-FOXO1 group, MMP13 and ADAMTS-5 were attenuated while Collagen II and Aggrecan were increased. Co-transfection of MEG3 and si-FOXO1 eliminated the influence of si-FOXO1 (Fig. 5e) (*P* < 0.05). Taken together, these data demonstrated that FOXO1 can strengthen the effects of MEG3 in OA.

### MEG3 relieved the cartilage matrix degradation in OA rats

**Fig. 6:**
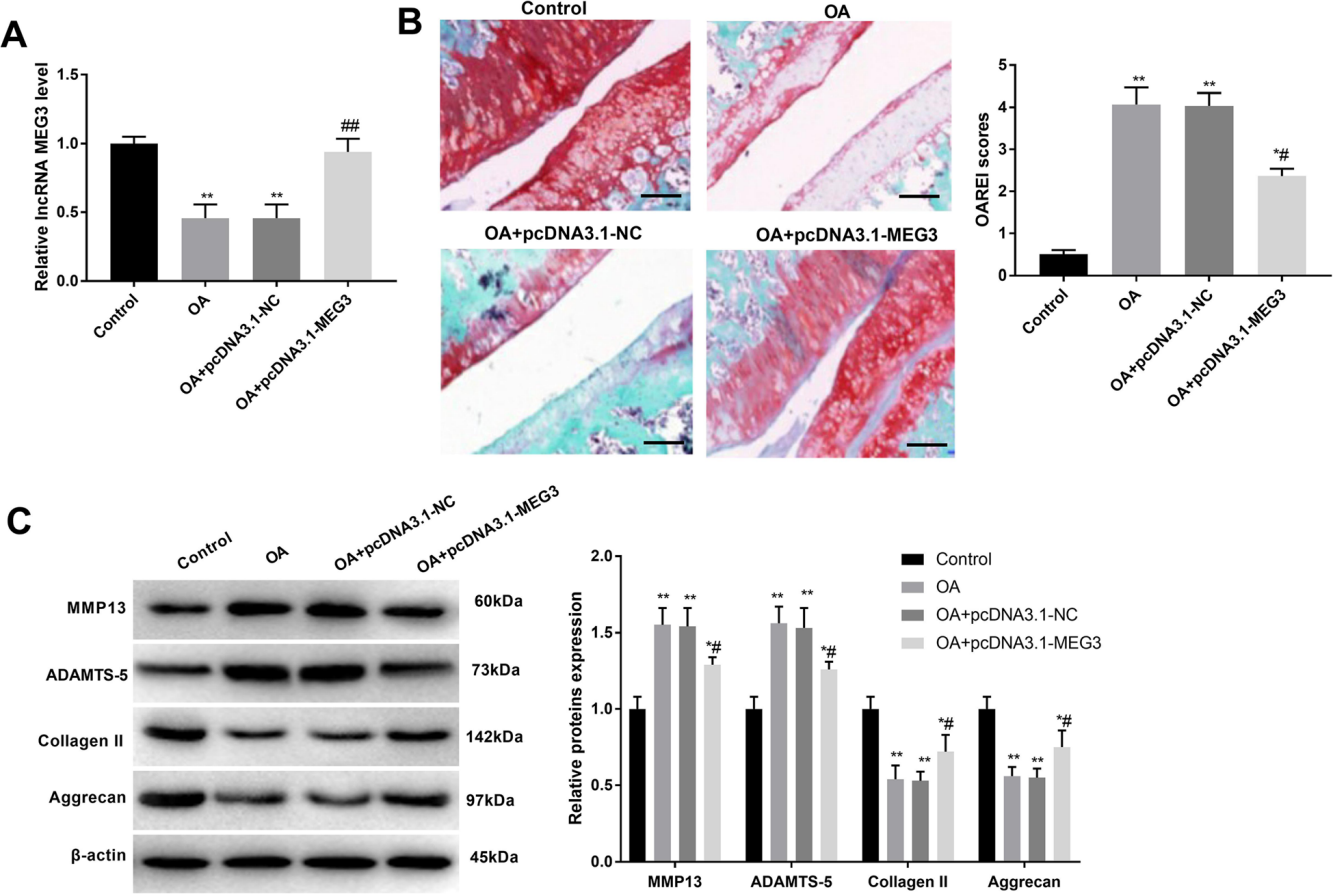
MEG3 relieved cartilage matrix degradation in OA rats. **a**, MEG3 expression in cartilage tissue samples of each group. **b**, Safranin O staining and OARSI grade (Bar = 100 μm) of cartilage tissue samples from each group. **c**, the expression of MMP13, ADAMTS-5, Collagen II and Aggrecan proteins in rat cartilage tissue samples. *, *P* < 0.05 when compared with the control group; ^#^, *P* < 0.05 when compared with the OA + pcDNA3.1-NC group

To ensure the successful delivery of pcDNA3.1-NC or pcDNA3.1-MEG3 into the chondrocytes, we measured the MEG3 levels and the results showed that pcDNA3.1-MEG3 had been successfully delivered into OA rats (Fig. 6a) (*P* < 0.01). Injection of pcDNA3.1-MEG3 effectively reduced the cartilage damage of the operation, protected the cartilage from degradation, and reduced the loss of proteoglycan and joint soft cell (Fig. 6b) (*P* < 0.01). Moreover, based on the results of ECM degradation-related protein expression levels, the interference of pcDNA3.1-MEG3 inhibited cartilage bone marrow matrix degradation (Fig. 6c) (*P* < 0.05). These findings demonstrated that MEG3 could attenuate the degradation of ECM in OA rats.

## Discussion

Although OA affects about 237 million people worldwide [19], the molecular mechanism of OA has not yet been investigated in detail. In the current study, qRT-PCR analysis revealed that MEG3 expression was significantly inhibited in OA, while miR-361-5p expression increased. On the basis of a StarBase prediction, we showed that MEG3 was a binding partner of miR-361-5p in OA chondrocytes. In addition, functional experiments showed that MEG3 can promote cell proliferation and inhibit cell apoptosis through interacting with miR361-5p.

As a maternally expressed imprinted gene, MEG3 has been found in various normal tissues (except for tumor cells) [11, 20]. A previous study showed that down-regulation of MEG3 was associated with the expression of tumor genes in epithelial cells [21]. Ying et al. showed that suppression of MEG3 was related with the progression of bladder cancer [22]. In an earlier publication, low MEG3 expression was shown to inhibit apoptosis of chondrocytes in the OA rat model [23]. Low expression of MEG3 has already been demonstrated in OA. MEG3 has been shown to participate in the development of OA via miR-16/AMAD7 interactions [15]. MEG3 was down-regulated in OA and inversely associated with vascular endothelial growth factor A levels [13]. In the current study, qRT-PCR analysis showed that the expression levels of MEG3 in the OA group were significantly lower than in normal group. Thus, we speculated that MEG3 might be involved in the progression of OA. In an attempt to understand the role of MEG3in OA, we performed several functional experiments and found that MEG3 promoted cell viability while inhibited apoptosis in OA. OA is a severe malignancy characterized by ECM degradation. Enhancement of ECM degradation can accelerate the progression of OA [24]. The ECM is mainly composed of Collagen II and Aggrecan, which were degraded by collagen-specific proteases, such as matrix metalloproteinases (MMPs) [25]. MMP-13, a key collagenase in OA, shows highly up-regulated expression in OA [26, 27]. In addition, inhibition of ADAMTS-5 can relieve the symptoms of OA [28]. Herein, to further assess the effects of MEG3 in OA development, the expression of several protein factors related to ECM degradation were analysed by western blot. The results revealed that MEG3 suppressed the degradation of ECM. Thus, MEG3 may protect chondrocytes from several damaging effects of OA.

Extensive evidence suggested that miRNAs play a crucial role in OA progression. Wang et al. demonstrated that miR-140-5p played a role in chondrocyte proliferation, apoptosis, and inflammation in OA [29]. miR-203 inhibition could ameliorate OA cartilage degradation in a postmenopausal rat model [30]. We used StarBase to predict the target miRNA of MEG3 and found that miR-361-5p was a target of MEG3. There have been numerous reports in the literature indicating that miR-361-5p can participate in multiple tumors, such as gastric cancer [31], liver cancer [32], cholangiocarcinoma [33] and so on. However, the role of miR-361-5p in OA has not been elucidated. The present study demonstrated that miR-361-5p was highly regulated in OA. Furthermore, miR-361-5p can reverse the regulation of cell viability, apoptosis, and ECM degradation caused by MEG3 in OA.

Previous studies have reported that the FOXO proteins are an evolutionarily conserved family of transcription factors that exert important effects during development, aging, and longevity. This family consists of four members, including FOXO1 [34, 35]. More importantly, silencing SGK1 can alleviate the chondrocyte anabolic and catabolic imbalance through stimulating FOXO1-mediated autophagy in human chondrocytes [36]. Levinger et al. also detected the levels of FOXO1 in skeletal muscle, serum and synovial fluid in patients with knee OA [37]. A bioinformatics analysis in our present study suggested that FOXO1 might serve as a target gene of miR-361-5p; FOXO1 is negatively modulated by miR-361-5p and positively regulated by MEG3. In concert with MEG3, FOXO1 elevated cell proliferation, hindered apoptosis, and reduced the degradation of ECM. To summarize these interactions, a schematic of the expressional patterns of factors in the MEG3/miR-361-5p/FOXO1 axis and its regulatory effects on OA is showed in Additional file 1: Figure S1.

## Conclusions

In conclusion, MEG3 can positively modulate FOXO1 by binding miR-361-5p in OA. Furthermore, MEG3 may promote cell proliferation, impair cell apoptosis, and reduce ECM degradation via the miR-361-5p/FOXO1 axis in OA chondrocytes. These data may shed new insights into the underlying mechanisms of OA and identify novel targets for the treatment of OA.

## Supplementary information

**Additional file 1:** **Figure S1.** A schematic with respect to the expression of MEAG3/miR-361-5p/FOXO1 and their regulation on OA. +, represents an increase; −, represents a decrease.

## Data Availability

The datasets analyzed in the present study are available at https://www.ncbi.nlm.nih.gov/search/ with accession number Gene ID55384(ENSG00000214548; http://asia.ensembl.org/Homo_sapiens/Gene/Sequence?g=ENSG00000214548;r=14:100779410-100861031), Gene ID17263 (ENSMUSG00000021268; http://asia.ensembl.org/Mus_musculus/Gene/Sequence?g=ENSMUSG00000021268;r=12:109541001-109571726), Gene ID2308 (ENSG00000150907; http://asia.ensembl.org/Homo_sapiens/Gene/Sequence?g=ENSG00000150907;r=13:40469953-40666641), and Gene ID494323 (ENSG00000199051; http://asia.ensembl.org/Homo_sapiens/Gene/Sequence?g=ENSG00000199051;r=X:85903636-85903707;t=ENST00000362181). The other datasets analyzed in this current study are available from the corresponding author on reasonable request.

## Abbreviations

lncRNA: Long noncoding RNA
lncRNAs: Long noncoding RNAs
MEG3: Maternally expressed 3
OA: Osteoarthritis
