# Supplementary figures and images for: MEG3 promotes proliferation and inhibits apoptosis in osteoarthritis chondrocytes by miR-361-5p/FOXO1 axis
Source: BMC Med Genomics. 2019 Dec 30;12:201. doi: 10.1186/s12920-019-0649-6 (PMC6937924; doi:10.1186/s12920-019-0649-6)

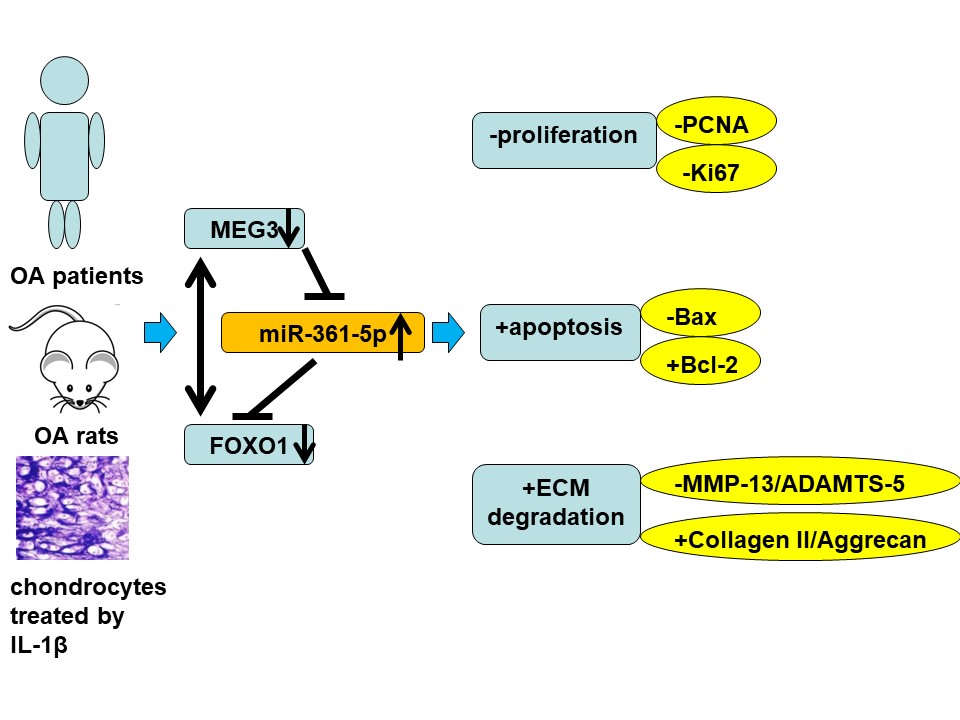

Supplement: Supplementary file 1 — Additional file 1: Figure S1. A schematic with respect to the expression of MEAG3/miR-361-5p/FOXO1 and their regulation on OA. +, represents an increase; −, represents a decrease. [file 12920_2019_649_MOESM1_ESM.jpg]
